# Taking Sides: An Integrative Review of the Impact of Laterality and Polarity on Efficacy of Therapeutic Transcranial Direct Current Stimulation for Anomia in Chronic Poststroke Aphasia

**DOI:** 10.1155/2016/8428256

**Published:** 2015-12-27

**Authors:** Margaret Sandars, Lauren Cloutman, Anna M. Woollams

**Affiliations:** Neuroscience and Aphasia Research Unit, School of Psychological Sciences, 3rd Floor, Zochonis Building, University of Manchester, Brunswick Street, Manchester M13 9PL, UK

## Abstract

Anomia is a frequent and persistent symptom of poststroke aphasia, resulting from damage to areas of the brain involved in language production. Cortical neuroplasticity plays a significant role in language recovery following stroke and can be facilitated by behavioral speech and language therapy. Recent research suggests that complementing therapy with neurostimulation techniques may enhance functional gains, even amongst those with chronic aphasia. The current review focuses on the use of transcranial Direct Current Stimulation (tDCS) as an adjunct to naming therapy for individuals with chronic poststroke aphasia. Our survey of the literature indicates that combining therapy with anodal (excitatory) stimulation to the left hemisphere and/or cathodal (inhibitory) stimulation to the right hemisphere can increase both naming accuracy and speed when compared to the effects of therapy alone. However, the benefits of tDCS as a complement to therapy have not been yet systematically investigated with respect to site and polarity of stimulation. Recommendations for future research to help determine optimal protocols for combined therapy and tDCS are outlined.

## 1. Introduction

Aphasia is an acquired disorder that affects the way in which an individual produces and/or understands language [1]. Language is an essential aspect of communication and aphasia can impact significantly on the daily functioning and quality of life of stroke survivors [2]. The neural network supporting speech production is extensive [3] and hence easily disrupted by damage, such as a stroke. It is therefore perhaps unsurprising that anomia, or word finding difficulty, is the most common and persistent symptom across all types of aphasia [4]. Indeed, those with more severe acute deficits tend to recover to this level [5] and, consequently, amelioration of anomia is a frequent aim in poststroke rehabilitation. The typical approach to the treatment of anomia is impairment-based behavioral speech and language therapy, which focuses on helping the patient to “relearn” words they are unable to retrieve or produce. This type of therapy can improve both object naming [6] and everyday communicative abilities [7, 8]. Yet it can be time-consuming to even achieve small gains. Consequently, researchers have begun to investigate more innovative new treatments based on neuroscientific principles. Recent research has suggested that neurostimulation techniques, such as transcranial Direct Current Stimulation (tDCS), can be used to optimize therapeutic gains.

The purpose of this review is to evaluate current research on the use of tDCS in the treatment of chronic poststroke anomia to determine what has been learnt so far regarding its application and efficacy, with particular reference to the important factors of polarity (whether stimulation is positive or negative) and site of stimulation (notably, left hemisphere versus right hemisphere). Critical gaps in the literature are identified, and recommendations for future research into this combined therapeutic approach are outlined. In contrast to previous reviews on this topic (e.g., [9–14]), the present review will specifically focus on studies that have examined the effects of tDCS on confrontation naming of noun and verb pictures in chronic aphasia via a range of research designs, with reference to current neuroscientific models of speech processing and aphasia recovery.

## 2. Naming and Recovery

### 2.1. The Neural Naming Network

Models of language production propose that a number of interrelated tasks are necessary in order to produce speech, involving processing at semantic, phonological, and articulatory levels [15, 16]. Thus, some models of confrontation naming propose that, when presented with a picture of an object and asked to state the object's name, individuals must first map the visual stimulus onto a stored conceptual representation of the object (visual object recognition and semantic access), then retrieve its name (lexical retrieval) and phonological form (phonological code retrieval and phonological encoding), and create a phonetic representation of the name (phonetic encoding), before generating a motor articulatory sequence of the phonetic representation for the vocal tract to follow (articulation) [15, 17].

The brain areas believed to be involved in normal speech comprehension and production have been conceptualized within the dual-stream framework proposed by Hickok and Poeppel [3, 18]. A version of this framework has also been implemented as a neuro-computational model by Ueno et al. [19]. According to the dual stream model, two distinct pathways link language-related regions: the dorsal stream and the ventral stream. The left-dominant dorsal stream is primarily responsible for mapping sensory input and phonological information onto the articulatory network. This pathway extends anteriorly from area Spt (a left-dominant area in the planum temporale, named according to its location in the Sylvian fissure at the parietotemporal boundary) via the arcuate fasciculus to the posterior inferior frontal gyrus (IFG, including Broca's area), the anterior insula, and areas of the premotor cortex. The ventral stream consists predominantly of bilateral structures in the posterior and anterior parts of the temporal lobes surrounding the middle temporal gyrus (MTG) and inferior temporal sulcus (ITS). Both the dorsal and ventral pathways are linked to other cortical areas that play important roles in speech and language tasks, including the bilateral superior temporal gyrus (STG), superior temporal sulcus (STS), and areas of the frontal cortex. The left STG and ventral stream structures incorporate what is commonly referred to as Wernicke's area [20]. The role of the ventral stream is mapping sounds onto meanings and meanings onto spoken output. Consequently, the ventral stream is believed to be involved in a variety of semantically mediated tasks, including auditory comprehension and picture recognition. Consequently, oral picture naming relies on elements of both the dorsal and ventral streams.

Research has shown that naming, alongside other speech production tasks, is typically lateralized to the left hemisphere in healthy individuals [21]. More specifically, neuroimaging studies of healthy adults have shown picture naming to be associated with left lateralized activation in the MTG, posterior STG, thalamus, posterior IFG (namely, pars opercularis, BA44, pars triangularis, BA45 and BA46) [22–24]. When the naming context is manipulated to make word finding more or less demanding, additional regions are recruited in both hemispheres, such as the bilateral fusiform gyri for less familiar items and the bilateral premotor cortex for items with longer names [25]. Imaging studies of stroke survivors also support the dual stream model. For example, Butler et al. [26] localized phonological and semantic deficits to damage to the dorsal and ventral pathways, respectively. More specifically, voxel-based lesion-symptom mapping (VLSM) studies have revealed that lesions to the left orbital IFG (BA47) and posterior MTG are significantly correlated with impaired picture naming [27] and, correspondingly, that lack of damage to the left midposterior MTG and underlying white matter tracts is critical for successful oral picture naming [28]. Piras and Marangolo [29] further highlighted the complexity of the neural network underpinning naming. In their study, impaired noun naming was associated with lesions to the left STG and MTG, while impaired verb naming was more strongly associated with a wider range of lesion sites, extending from BA45 to the anterior temporal lobe (BA22, BA38).

### 2.2. Language Recovery

Despite damage to language processing areas, most individuals who have suffered a left hemisphere stroke are able to recover at least some language skills, both spontaneously and following therapy, even many years after onset [30]. Language recovery following stroke can be considered to take place during three overlapping temporal stages: acute (hours to days), subacute (weeks to months), and chronic (months to years) [30]. This recovery is facilitated by several different mechanisms that play key roles during different stages, such as the restoration of blood flow during the acute stage (e.g., [31, 32]), the functional recovery of intact, temporarily dysfunctional brain regions during the subacute stage (e.g., [33]), and the brain's ability to undergo significant structural and functional reorganization following damage, that is, neuroplasticity, well into the chronic stage.

#### 2.2.1. Neural Regions Associated with Spontaneous Recovery

Researchers have attempted to explore the evolution of changes in spontaneous (re)organization of language function within the brain, particularly in relation to the relative influence of the impaired left hemisphere versus the intact right hemisphere. Saur and colleagues [34] found that different temporal stages were associated with different patterns of cerebral activation. In their longitudinal study, participants were scanned using fMRI and completed an aphasia test battery at three points (acute: 0–4 days, subacute: 2 weeks, and chronic: 4–12 months after onset) during their first year after stroke. Compared to age-matched controls, the stroke survivors showed reduced activation in the left IFG during the acute stage, with better initial language performance correlated with higher activation in this region. In contrast, two weeks later, strong bilateral activation was observed, and early relative improvement in language abilities was associated with increased activation in regions within the* right* IFG and adjacent insular cortex and the right supplementary motor area. At the final assessment point, however, language activation had shifted back to areas including the left IFG and MTG was associated with further, significant improvement in language abilities.

The precise timings of changes in hemispheric dominance may vary between individuals (e.g., [35]). Nevertheless, this sequence of brain reorganization is supported by a recent review by Anglade et al. [36], and research confirms that, by the chronic stage, stroke survivors with the most favorable language recovery appear to be those who, like healthy individuals, demonstrate predominantly left lateralized language functions (e.g., [37]). When critical left hemisphere language areas are irretrievably damaged, compensatory recruitment of undamaged regions immediately surrounding the damaged areas (“perilesional” areas) is consistently linked to improvement in language abilities in chronic aphasia [38]. For example, Fridriksson et al. [39] found that stroke survivors with better naming ability showed greater activation than both control participants and patients with poorer naming ability in areas perilesional to Broca's area, including BA32 (anterior cingulate gyrus) and BAs 10 and 11/47 (medial and middle frontal gyrus). The role of the right hemispheric activation in the chronic stage remains more controversial [40]. One theory maintains that damage to the left hemisphere can lead to transcallosal disinhibition, meaning that homologous areas in the right hemisphere that are normally inhibited by the left during language tasks become overactive and, in turn, may impose greater inhibition on the left hemisphere language regions [41]. In support of this hypothesis, a number of fMRI studies have shown that individuals with chronic poststroke aphasia do indeed have higher activation in areas such as the right IFG and right STG than healthy controls when carrying out a range of language tasks (e.g., [42, 43]). Activation in the right IFG has, however, been associated with errors of omission and semantic paraphasias in picture naming [4]. One potential explanation for such findings is that hyperactivation in the right hemisphere may prevent recruitment of perilesional areas in the left hemisphere, hindering long-term recovery from aphasia [44].

#### 2.2.2. Neural Regions Associated with Therapeutic Recovery

Further neuroimaging studies indicate that speech and language therapy can facilitate recruitment of perilesional language areas in the left hemisphere (such as the left precentral and supramarginal gyri) in individuals with chronic poststroke aphasia, resulting in improved oral picture naming ability and a reduction in both semantic and phonological errors [45–49]. In contrast, those who respond less favorably to therapy tend to activate a greater number of diverse areas in the left and right hemispheres during naming tasks [45]. Like spontaneous relateralization, left hemisphere rerecruitment following anomia therapy is likely to be a dynamic process. For instance, Menke et al. [50] found that, immediately following a computer-based intervention program, correct naming was related to increased bilateral and right hemisphere activity in regions including the bilateral parahippocampal gyri, right precuneus, cingulate gyrus, and both occipital lobes. However, by eight months after therapy, as naming ability was consolidated, success on trained items was associated with increased activity in left perilesional middle and superior temporal areas, along with some increased activity in the right hemisphere Wernicke's homologue. The authors suggest that the residual right hemisphere activity at eight months after therapy could have been functionally beneficial for particular individuals in their study, who had large left hemisphere lesions that made full left relateralization of language function unfeasible (see also [51]).

To conclude, stroke survivors with damage to the left hemisphere may activate homologous areas in the right hemisphere in order to recapture some degree of language ability at varying stages in the recovery process. In the longer term, this is likely to be a less effective strategy than recruitment of perilesional areas in the left hemisphere, with research strongly suggesting that left hemisphere relateralization (as far as possible) is most beneficial for language recovery [51]. Behavioral speech and language therapy can increase activity in the left hemisphere, and such activation is associated with superior outcomes from a variety of poststroke treatment programs. However, all these studies have incorporated intensive treatment protocols, which are not always available in clinical settings and do not suit all patients [12]. Consequently, researchers have begun to investigate the potential of neurostimulation techniques, namely, Transcranial Magnetic Stimulation (TMS) and transcranial Direct Current Stimulation (tDCS), to facilitate the language recovery process.

## 3. Neurostimulation to Enhance Recovery

### 3.1. Transcranial Magnetic Stimulation (TMS)

TMS involves the delivery of rapidly alternating magnetic fields to the underlying cortical tissue via an electromagnetic coil placed on the scalp. The effects of TMS vary according to the frequency of electromagnetic pulses. High frequency, or fast, TMS (≥5 Hz) can induce increases in cortical excitability. In contrast, low frequency, or slow, TMS (typically 1 Hz) is associated with cortical inhibition [14]. The majority of studies investigating the therapeutic effects of TMS on poststroke anomia have involved the application of low frequency TMS to the right hemisphere. This is based on the rationale discussed above that language deficits persist due to right hemispheric inhibition of perilesional left hemisphere language regions [10]. Consequently, inhibiting this inhibition via the application of TMS should theoretically lead to improvements in naming ability.

In support of this theory, Naeser and colleagues [52, 53] demonstrated, across a series of studies, that applying repetitive slow (inhibitory) TMS to the right hemisphere of patients with chronic aphasia had beneficial effects on their language skills. In the first study, three nonfluent participants all with lesions involving damage to Broca's area received single ten-minute sessions of 1 Hz TMS either in the right Broca's homologue (pars triangularis, BA45) or in the mouth area of the motor cortex [52]. The researchers found that only stimulation to the pars triangularis portion of the right Broca's homologue significantly increased picture naming accuracy, thus supporting the notion that dysfunctional right hemisphere overactivation had previously been adversely affecting naming skills. These effects were, however, short-lived and disappeared within 30 minutes. In an attempt to produce longer lasting effects, the same research group administered 1 Hz TMS to the pars triangularis of the right Broca's homologue of four stroke survivors (two with Broca's aphasia, one with Broca's aphasia recovered to Anomic/Conduction aphasia, and one with Global aphasia) for 20 minutes a day, five days a week, for two weeks [53]. Language abilities were assessed at baseline and again at two weeks, two months, and eight months after TMS. As in Naeser et al.'s earlier study, TMS resulted in significantly better naming ability for all four participants, this time in terms of both naming accuracy and speed. Furthermore, for three of the four participants, these effects were maintained for eight months following stimulation. This suggests that multiple stimulation sessions led to long-term brain reorganization, although the authors did not use brain imaging tools to confirm this hypothesis.

One criticism of Naeser et al.'s studies is that all participants received only active TMS. Although unlikely, it is possible that the observed effects on naming abilities were not the direct result of the suppression of right hemispheric activation, but due to an unidentified factor related to the presence of the TMS equipment. To clarify this issue, Barwood and colleagues [54] recruited a dozen individuals with long-standing aphasia of varying severities. Half of the participants received 1 Hz TMS to the right pars triangularis, while the other half acted as a control group, receiving sham stimulation instead. Only active stimulation resulted in significant increases in naming accuracy and speed both immediately and one week after the stimulation sessions, thus supporting the view that inhibition of right hemisphere activation was responsible for improvements at single word production level.

The results of the TMS studies outlined above suggest that poststroke language production skills are optimized when activation in right frontal regions (and in particular the right pars triangularis) is reduced. However, as is the case with spontaneous recovery, individual differences play a significant role in a person's potential for language recovery following TMS. Factors shown to influence language recovery in aphasia include lesion site, lesion size, age, gender, handedness, and premorbid intelligence levels [55]. The particular importance of lesion site was demonstrated by Martin et al. [41], who administered ten sessions of slow TMS to the right pars triangularis of two individuals with chronic, nonfluent aphasia. Patient 1 (P1) responded well behaviorally to the TMS treatment. He named more object pictures and used longer phrases during an elicited speech task 3, 16, and 46 months after TMS than he had done before. In line with these increases in language performance, P1 also showed increased left hemisphere activation in perilesional sensorimotor cortical regions following TMS. In contrast, TMS had no significant effects on P2's measured language abilities. Nor did he demonstrate any new and lasting perilesional activation in the left hemisphere after stimulation. The authors suggest that the differences in response to TMS between P1 and P2 were likely to be related to their lesion sites. While both participants had lesions to Broca's and Wernicke's areas, unlike P1, P2 had additional lesions in the left motor and prefrontal cortices and regions both inferior and posterior to Wernicke's area. The additional left hemispheric damage to P2's extended language network may have prevented him from activating perilesional areas following inhibitory TMS to the right hemisphere.

In each of the studies above, participants received only low frequency TMS in isolation. It is possible that administering TMS followed by behavioral speech and language therapy may be more efficient than either TMS or therapy alone in increasing language abilities in individuals with aphasia [56]. To examine the potential enhancing effect of TMS on speech and language therapy, Weiduschat and colleagues [57] applied up to 1 Hz low frequency TMS to either the right pars triangularis or the vertex (as a sham condition) of small groups of subacute stroke survivors with different types of aphasia, five days a week for two weeks. In each session, 20 minutes of stimulation was immediately followed by 45 minutes of individually tailored speech and language therapy. Results showed that while language abilities including single word naming increased in both groups of participants after intervention, this increase was only significant for the participants who had received TMS to the right pars triangularis. This finding indicates that therapy sessions that combine inhibitory right hemisphere TMS with more traditional speech and language therapy can result in greater therapeutic gains when compared to therapy alone, at least for subacute stroke survivors. Other research suggests that combining enhancing activity in the left hemisphere via excitatory TMS with speech and language therapy can also convey therapeutic benefits. For instance, Cotelli et al. [56] gave three patients with chronic aphasia 25 minutes of high frequency TMS to the left dorsolateral prefrontal cortex, immediately followed by 25 minutes of therapy designed to increase noun naming ability. TMS targeted a region whose excitatory stimulation has been shown to facilitate naming in both healthy controls [58] and individuals with Alzheimer's disease [59]. All patients received at least a fortnight of real TMS plus therapy. In line with expectations based on these previous findings, two weeks of combined TMS and anomia therapy led to significant improvements in the percentage of correctly named objects. This effect generalized to untreated items and persisted for both treated and untreated items until the final follow-up, 48 weeks after intervention.

In summary, applying low frequency TMS to the right hemisphere or high frequency TMS to the left hemisphere appears to have some therapeutic benefit for individuals with subacute or chronic poststroke anomia, whether administered alone or in conjunction with behavioral speech and language therapy. More research is required to tease out the relative effects of TMS and behavioral therapy. However, the practical appeal of TMS as a therapeutic tool is somewhat limited. For instance, TMS can cause muscle twitching which, as well as being unpleasant for patients, may hinder verbal responses if their facial muscles are affected [60]. Additionally, the noise of the stimulator may make it difficult for patients to complete therapy tasks. Consequently, it is not generally feasible to apply TMS concurrently with behavioral speech and language therapy or create effective sham conditions. To overcome these issues, research has increasingly focused on an alternative technique that shows particular promise as a therapeutic tool, transcranial Direct Current Stimulation (tDCS) [61].

### 3.2. Transcranial Direct Current Stimulation (tDCS)

tDCS is a noninvasive neurostimulation technique that uses a battery pack to deliver weak electrical currents to the brain via two saline-soaked electrodes. The active electrode is placed on the scalp over a particular region of interest, stimulating the cortex underneath, while the reference electrode is usually placed on the contralateral supraorbital or contralateral shoulder [48]. Positive (anodal) stimulation is associated with increased neuronal excitability while negative (cathodal) stimulation is associated with inhibition of neuronal activity [62].

#### 3.2.1. Neurobiology of tDCS-Induced Excitability Changes

Research has shown that the effects of tDCS on brain activation and task performance are determined by multiple factors, including the number of stimulation sessions, the strength, and duration of the current applied, as well as the task in hand [63]. After effects have been found to be potentially long-lasting, persisting up to twelve months after stimulation [64], the physiological mechanisms underlying the effects of tDCS are not yet fully understood. However, unlike TMS, the currents generated by tDCS are considered insufficient to directly induce action potentials [14], and different processes are believed to be responsible for changes in cortical activation during and after stimulation [61]. During stimulation, tDCS is thought to indirectly alter neuronal excitability by temporarily affecting membrane polarity: anodal stimulation causes neuronal depolarization (increased sodium and calcium ion channel activity), whereas cathodal stimulation causes neuronal hyperpolarization (decreased sodium and calcium ion channel activity) [65, 66]. This proposition is supported by the observation that blocking sodium channels (using carbamazepine, or CBZ) and calcium channels (using flunarizine, or FLU) prior to stimulation reduces the excitatory effects of anodal tDCS, but it does not impact the effects of cathodal stimulation [65, 66].

While the short-term effects of tDCS appear to rely on transient changes in membrane potential, poststimulation effects are believed to be the result of longer-lasting changes in synaptic strength [61]. One likely mechanism by which tDCS may act to modulate synaptic strength is long-term potentiation (LTP). LTP is based on the Hebbian principle [67] that when pre- and postsynaptic neurons repeatedly fire together, metabolic changes occur which make the firing of one neuron more likely to result in the firing of the other in future. The result of LTP (and its reverse process, long-term depression, or LTD) is stable changes in synaptic activation that persist over many months or even years [68]. The inducement of LTP or LTD is dependent on the levels of specific neurotransmitters and neuromodulators (neurochemicals that can potentiate or attenuate the responses evoked by neurotransmitters) [63]. In particular, tDCS appears to involve the regulation of the excitatory neurotransmitter glutamate and the inhibitory neurotransmitter GABA, plus the neuromodulators dopamine, acetylcholine, and serotonin [61]. To examine the relationship between tDCS and changes in cortical neurotransmitter concentrations, Stagg and colleagues [69] administered 1 mA of anodal, cathodal, and sham tDCS to the left primary motor cortex of 11 healthy adults in three separate sessions, at least seven days apart, and examined the effects using magnetic resonance spectroscopy (MRS). These MRS results showed that anodal stimulation led to significant decreases in GABA concentration. In comparison, cathodal stimulation led to significant decreases in glutamate levels as well as correlated decreases in GABA concentration. This latter finding may initially appear at odds with expectations; however, GABA is synthesized from glutamate and, therefore, reducing the amount of available glutamate via inhibitory tDCS will result in corresponding decreases in GABA [69]. Taken together, Stagg et al.'s results indicate that the after effects of anodal tDCS are mediated, at least in part, by a reduction in GABAergic inhibition, while the after effects of cathodal stimulation are related to a reduction in glutamatergic neurotransmission. Other researchers have shown that, as well as glutamate and GABA themselves, NMDA receptors also play an important role in the development of tDCS-induced after effects. For example, Nitsche and colleagues [65, 66] demonstrated that administration of the glutamate antagonist dextromethorphan (DMO), which acts to block NMDA glutamate receptors, abolished the after effects of both anodal simulation and cathodal stimulation.

With respect to neuromodulators, acetylcholine has been found to have an adverse impact on potential tDCS-induced alterations in neuronal excitability. In one study, increasing acetylcholine levels by administering the acetylcholinesterase inhibitor rivastigmine eliminated the after effects of anodal tDCS and reduced the after effects of cathodal tDCS [70]. In comparison, increasing serotonin levels via the use of the selective serotonin reuptake inhibitor citalopram both enhanced and prolonged the excitatory after effects of anodal tDCS and reversed the inhibitory after effects of cathodal tDCS to produce excitation [71]. Conversely, increasing dopamine via its precursor L-DOPA turned anodal tDCS-induced excitability to inhibition and extended cathodal tDCS-induced reductions in excitability by several days [72]. Thus, serotonin appears to facilitate excitatory stimulation while dopamine facilitates inhibitory stimulation. However, the impact of neuromodulator levels on tDCS effects is complex, and they do not appear to follow simple, linear relationships. For example, in a study examining the influence of dopamine on cathodal after effects, Monte-Silva and colleagues [73] found that only intermediate doses (0.5 mg) of ropinirole (a D_2_ dopamine receptor agonist) increased the inhibitory after effects of cathodal tDCS, with low (up to 0.25 mg) and high doses (1.0 mg) actually abolishing the effects instead. Further investigation is required to clarify the intricate interactions between neurotransmitters and neuromodulators in inducing and sustaining the behavioral effects of tDCS.

An important caveat to acknowledge regarding the use of tDCS is that applying an electrical current to the brain transcranially (as opposed to directly stimulating the cortex) may mean that the underlying cortex fails to receive the expected dose of stimulation, resulting in the recipient failing to demonstrate the desired behavioral consequences. One reason for this is the dispersion of current before it reaches the target cortex. For example, Miranda et al. [74] modelled the spatial distribution of 2 mA anodal tDCS delivered to four different cortical regions. Their results revealed that the intensity of current on the scalp directly underneath the anode varied, in that the current density was observed to be higher at the perimeters than in the center of the electrode. Although current density was more uniform once it reached the brain surface, between 41% and 61% of the current did not penetrate through the skull to the cortex underneath. Research has also revealed that, even once current reaches the cortex, the effects of tDCS on brain activity may not be restricted to areas directly under the active electrode, but they can extend to a wider network of functionally related brain regions via excitatory and inhibitory neural pathways [75]. For instance, in one study, anodal tDCS to the dorsal lateral prefrontal cortex of ten healthy volunteers led to increased synchronous activity between distal frontal and parietal areas [76]. Finally, it is important to note that studies that have examined the neurobiological basis of tDCS have generally only considered its effects on healthy humans, or even on animal subjects. It is possible that the neurological activation patterns and subsequent behavioral effects may not be the same in stroke-damaged human brains as they are in healthy ones [77]. In support of this, Datta et al. [78] modelled the current flow as a result of anodal stimulation to the left frontal cortex (BA6) in a nonfluent patient who had responded favorably to an intervention program combining tDCS and computerized anomia therapy. Their analysis revealed that current flow in this particular individual was indeed altered from the pattern observed in a healthy brain due to the presence of the lesion, with the current found to be most concentrated in deep, perilesional brain regions. Furthermore, they observed that current flow was also influenced by the positioning of the reference cathode, with different electric fields associated with contralateral shoulder, contralateral mastoid, contralateral supraorbital, and contralateral cortical homologue cathodes. As such, all of these factors should be borne in mind when designing protocols that aim to modify individuals' behavior with tDCS.

#### 3.2.2. Potential Advantages of tDCS as a Therapeutic Tool

Despite the caveats noted above, a growing body of evidence indicates that tDCS can have significant positive behavioral effects on a wide variety of cognitive and motor tasks in both healthy individuals and stroke survivors (e.g., [79–82]). From a practical viewpoint, tDCS has a number of key characteristics that make it a viable therapeutic tool for use within the poststroke population. tDCS is considered safe when administered in accordance with the established conventions and, unlike TMS, it is not associated with an increased seizure risk [65, 66, 83–85]. It is generally well tolerated, although individuals undergoing tDCS occasionally report side effects such as localized tingling, itching, burning, pain, and headaches, related to stimulation itself and to the bands used to hold electrodes in position. These effects are typically mild and fade within 30 seconds to 1 minute of stimulation [86, 87]. Side effects can also be reduced by soaking the sponge electrodes in a 15–140 mM saline solution [88]. Moreover, studies have not found any physiological differences in participants' systolic and diastolic blood pressure, heart rate, or rated mood between stimulation and sham (no stimulation) conditions, further indicating the comfort and safety of tDCS [86, 89] and confirming that changes in arousal do not mediate the effects of tDCS on performance. Furthermore, as tDCS does not result in action potentials, it does not induce the muscle twitches associated with TMS. Taken together, these factors make tDCS an ideal method by which one can administer stimulation in conjunction with speech and language therapy, both “online” (with therapy and stimulation administered concurrently) and “offline” (with therapy following stimulation). The lack of physiological changes and the diminishing of the sensations associated with stimulation within one minute after onset also mean that recipients are often unable to distinguish sham (where active stimulation is administered for approximately 30 seconds to produce the initial sensations, before slowly being turned off) from longer periods of active stimulation (e.g., [86]). The potential to include this no stimulation control condition enables the studies to compare the effectiveness of behavioral speech and language therapy in conjunction with tDCS with that of behavioral speech and language therapy alone. Finally, tDCS equipment is relatively inexpensive and easily portable, making it theoretically possible for clinicians to administer tDCS to people with aphasia in a variety of contexts, including patients' own homes [79].

## 4. Therapeutic Effects of tDCS on Naming Ability in Aphasia

In order to thoroughly assess the therapeutic effects of tDCS on the naming performance of individuals with chronic stroke-induced aphasia, comprehensive searches of databases and other sources were carried out at several time points to obtain details of all relevant studies. Electronic databases (CINAHL Plus, Medline, and PubMed) were searched periodically between July 2013 and October 2014 to identify possible papers, published in English in peer-reviewed journals. The search terms used were “tDCS,” “transcranial direct current stimulation,” “stimulation,” or “neurostimulation” in combination with “language,” “aphasia,” or “anomia.” Although broad, these search terms were chosen to maximize identification of all relevant studies. No specific publication dates were imposed. In addition, the “related citations” suggested by PubMed and the reference lists of relevant papers were also checked. All generated papers were then closely examined to confirm that they involved the use of tDCS rather than alternative brain stimulation techniques, such as TMS, and that any therapy provided and any outcome measures used focused primarily on single word confrontation naming of object and/or action pictures. Studies were only included if some or all of the participants were adult stroke survivors with chronic aphasia, meaning that studies that involved language production in healthy participants and/or stroke survivors in the acute or subacute stages alone were omitted [103–107].

Following the literature search, 14 studies that directly investigated the therapeutic effects of tDCS on single noun or verb picture naming in individuals with chronic poststroke aphasia, both as a stand-alone technique and in conjunction with behavioral speech and language therapy, emerged. These studies are summarized in Table 1. Studies are grouped by stimulation hemisphere: left, right, and bilateral, and their findings are discussed with reference to previously described TMS results.

### 4.1. Left Hemisphere Stimulation

Two studies investigated the effects of left hemisphere tDCS alone on naming ability in individuals with aphasia [90, 91]. In a preliminary study, Monti et al. [90] administered tDCS to eight chronic nonfluent aphasic individuals. In the first part of their study, all participants received one ten-minute session of sham tDCS to Broca's area. In addition, six participants received a further session of 2 mA anodal stimulation and six received a further session of 2 mA cathodal stimulation to Broca's area (four participants received all three types of stimulation). Picture naming was assessed before and immediately after each stimulation session. In the second part of the study, carried out two months later, all eight participants received single sessions of both cathodal and sham stimulations to the occipital lobe (2 cm above the inion). The results of both studies revealed that only cathodal tDCS to Broca's area significantly improved noun picture naming accuracy, which the authors attributed to a decreased excitability of inhibitory circuits within the left hemisphere. However, this result was obtained with a very limited sample size and, in contrast to studies showing the effectiveness of TMS alone in improving anomia [41, 52–54], other studies involving the application of tDCS to the left hemisphere in the absence of concomitant therapy tasks have shown little benefit, even when the overall dose of stimulation is greatly increased. For instance, within a diverse group of eight stroke survivors with chronic mild to moderate aphasia, Volpato and colleagues [91] demonstrated that, with the exception of one individual with severe anomia, 20 minutes of 2 mA anodal stimulation to Broca's area once a day for two weeks had no significant effects on either object or action naming.

In contrast to the application of tDCS alone, a number of studies have found evidence for the efficacy of anodal stimulation to the left hemisphere in conjunction with speech and language therapy in improving naming abilities in individuals with poststroke aphasia. For example, Baker et al. [92] gave ten patients with chronic stroke-induced aphasia (six fluent, four nonfluent) five consecutive days of anodal tDCS (1 mA for 20 minutes) and five consecutive days of sham tDCS. Participants completed a computerized matching task (following [108]) at the same time as receiving stimulation. This involved showing a series of color noun pictures, each immediately followed by an audio video clip of a man's mouth saying an object name. After each coupled presentation, patients were required to indicate whether the image and the associated video clip referred to the same item or not. Therapy runs were separated by a seven-day rest period to avoid carryover effects and the order of runs was counterbalanced across participants. During therapy, care was taken to ensure that the active electrode was placed over structurally intact perilesional cortex that had previously shown the most activation during a pretherapy naming assessment during fMRI. Consequently, electrode positioning varied slightly for each individual, although, across all participants, the active electrode was placed over either the left precentral gyrus or parts of the left frontal gyrus.

The study found that both the anodal and sham stimulation conditions resulted in increased numbers of correctly named treated items compared to baseline for the majority of participants. However, these increases were only significant in the anodal tDCS condition, with this effect maintained at follow-up, one week after therapy ceased. The number of correctly named untreated items also increased in the anodal tDCS condition, although this increase failed to reach statistical significance at either time point. More detailed inspection of Baker et al.'s results reveals that four participants (two fluent and two nonfluent) performed significantly better on the noun naming measure following anodal stimulation than following sham stimulation, indicating that they benefited more from active tDCS than the remaining six participants. This variability in therapeutic response was unrelated to aphasia severity. However, all four good responders had damage to the left frontal cortex, meaning that the perilesional stimulation was applied especially near to their lesion sites. It is possible that targeting intact tissue situated very close to damaged regions is critical to the effectiveness of tDCS as an adjunct to behavioral anomia therapy. Utilizing the same electrode positioning and therapy protocol as Baker et al. [92], Fridriksson et al. [93] showed that anodal tDCS plus computerized anomia treatment was significantly more effective in improving treated noun picture naming speed in a group of eight patients with chronic fluent aphasia, both immediately after treatment and at the three-week follow-up. Due to the location of their participants' lesions, the active electrodes were placed more posteriorly in Fridriksson et al.'s study than Baker et al.'s in order to stimulate regions close to Wernicke's area, again demonstrating the importance of proximal perilesional stimulation for maximal therapeutic outcomes. The results of these two studies also indicate that when used in conjunction with behavioral language therapy, anodal tDCS applied to intact perilesional cortical areas in the left hemisphere can benefit individuals with anomia associated with both fluent aphasia and nonfluent aphasia, demonstrating its wide clinical applicability.

The observation that anodal tDCS to the left hemisphere can enhance naming ability is further supported by four studies conducted by Fiori and colleagues [94, 95], Marangolo et al. [96], and Vestito et al. [97]. In the first of these studies, three individuals with chronic nonfluent aphasia completed two runs of therapy (each of five consecutive days), during which they were asked to name pictures of objects while receiving 20 minutes of 1 mA anodal or sham stimulation to Wernicke's area [94]. During therapy, written labels were provided when participants were unable to spontaneously name any item within 15 seconds. Results revealed that unsupported confrontation naming was faster and more accurate following anodal rather than sham stimulation. These observations held true for two individuals (one with moderate and one with severe nonfluent aphasia) who completed the final follow-up three weeks after therapy. More recently, Fiori et al. [95] extended their earlier work by investigating the effects of tDCS plus therapy on both noun naming and verb naming. Seven nonfluent patients took part in two three-week long therapy cycles, during which they received anodal stimulation to Broca's area, anodal stimulation to Wernicke's area, and sham stimulation over either Broca's (three participants) or Wernicke's (four participants) areas. Therapy involved individuals being asked to name depicted items or enacted actions that appeared on a computer screen, initially without cues. Objects and actions were matched for imageability, length, frequency, and age of acquisition. As in Fiori et al.'s previous study, in the event of failure to name the image within 15 seconds, participants were briefly presented with the written name. To minimize the potential impact of practice effects, the order of therapy cycles was counterbalanced across participants. The main finding from this study was an interaction between anodal stimulation location and lexical class in that tDCS to Broca's area significantly improved verb naming while tDCS to Wernicke's area significantly improved noun naming. These effects were still clearly evident at four weeks after therapy. Fiori et al.'s [95] findings are supported by a similar study carried out by Marangolo et al. [96] in which anodal tDCS to Broca's but not Wernicke's area was again associated with significant increases in verb naming accuracy for a diverse group of patients with nonfluent aphasia, both immediately after therapy and four weeks later.

Taken together, Fiori et al.'s [95] and Marangolo et al.'s [96] results indicate that the most effective site of stimulation depends on the lexical class of the treatment items. This finding is in line with VLSM work, linking noun naming to activity in the STG and MTG and verb naming to activity in the IFG and more anterior regions of the temporal lobe [29]. However, Vestito and colleagues [97] did not find the effects of frontal anodal stimulation to be qualified by lexical class. In their study, three individuals with nonfluent aphasia received 20 minutes of sham tDCS followed by 20 minutes of 1.5 mA anodal tDCS to Broca's area (with an hour's rest period between stimulation sessions) each weekday for a fortnight. Concurrently, with all tDCS sessions, participants were asked to name a total of 40 nouns and verbs in the absence of any cues or feedback. Separate treatment sets were used each week, with the second week incorporating increased numbers of lower frequency words in order to increase the task difficulty. Over both intervention weeks, the number of items correctly named by all participants increased significantly from baseline only following active stimulation. These significant effects were maintained for 16 weeks after stimulation and persisted, although they are no longer significant, until the final follow-up 5 weeks after this. Contrary to Fiori et al.'s and Marangolo et al.'s results, participants showed similar relative increases in both noun and verb naming following anterior stimulation.

The studies discussed above provide increasing evidence that combining anodal stimulation to the left hemisphere with concurrent speech and language therapy may significantly improve picture naming accuracy and/or speed in individuals with chronic anomia. This is in line with the findings obtained by Cotelli et al. [56], who noted that high frequency TMS to the left hemisphere facilitated correct noun naming in patients with chronic anomia for up to 48 weeks after therapy. In comparison, outcomes from unilateral left hemisphere tDCS studies have been maintained for up to 21 weeks after intervention, the longest follow-up reported. Stimulating both left frontal and temporal regions has been shown to be effective, with precise results likely to be dependent on individual patient characteristics, including lesion site, and also the word class targeted in therapy.

### 4.2. Right Hemisphere Stimulation

Akin to research into the therapeutic effects of TMS, studies have also investigated whether beneficial effects on naming may be obtained by using cathodal tDCS to inhibit supposedly dysfunctional activation in the right hemisphere and encourage left activation during language tasks. One such study was carried out by Kang et al. [98], who administered five consecutive days of 2 mA cathodal tDCS or sham tDCS to the undamaged right Broca's homologue of ten participants with differing aphasia diagnoses. Participants received 30 minutes of noun retrieval therapy each day, with tDCS applied for 20 minutes during each session. In line with previous TMS studies (e.g., [52–54]), Kang et al. found that cathodal stimulation was more effective than sham in increasing scores on a Korean version of the BNT [109], although this trend failed to reach statistical significance.

More recently, a larger, exploratory study carried out by Rosso and colleagues [99] reported significant increases in naming accuracy after lower intensity (1 mA) cathodal tDCS to the same right IFG site. Rosso et al. recruited 11 Anomic participants with lesions involving Broca's area (B+ participants) and 14 with lesions that left Broca's area intact (B− participants). All participants received single 15-minute sessions of both sham and cathodal tDCS to the undamaged right Broca's homologue, with the order of sessions counterbalanced across participants. Despite the facts that active and sham sessions were separated by only a two-hour washout period and patients did not complete a therapy task alongside stimulation, differences between conditions were significant. Results showed that changes in noun picture naming ability following cathodal tDCS were strongly related to lesion site in that naming accuracy of all B+ participants increased significantly while, for all but one of the B− participants, naming accuracy decreased or remained the same. This pattern of results is consistent with the notion that excessive inhibition by the undamaged right Broca's homologue on the damaged left hemisphere had been hindering naming abilities in the B+ participants until this inhibition was itself inhibited via cathodal stimulation (e.g., [10, 41]). Consequently, these findings support previous TMS studies in which inhibitory stimulation to the same cortical area significantly increased stroke survivors' naming abilities (e.g., [52–54]). Rosso et al. also discovered that individuals who demonstrated the greatest improvements in naming ability were those with the greatest integrity of the arcuate fasciculus, thus providing further support for the dual steam model and VLSM studies that posit Broca's area and the arcuate fasciculus as two neural components crucial for successful oral picture naming (e.g., [3, 27]).

Although Rosso et al. [99] did not include a concurrent therapy task, both this and Kang et al.'s [98] study suggest that cathodal stimulation to the undamaged hemisphere may be therapeutically beneficial for certain individuals with poststroke anomia. However, Kang et al. only collected outcome measures up to one hour after stimulation and Rosso et al. did not incorporate any follow-up period, making it impossible to know whether their interventions had any significant lasting effects, an important aim of most therapy programs. Furthermore, since cathodal tDCS to the right hemisphere was not compared to any other form of tDCS in either study, the relative effectiveness of each cannot be considered. In contrast, Flöel et al. [89] compared the effects of 1 mA anodal and cathodal applied tDCS to the right Wernicke's homologue of a mixed group of seven fluent and nonfluent participants while they carried out a computerized anomia therapy task. During therapy, participants were asked to name object pictures presented multiple times per session. Initially the pictures were shown alongside semantic, auditory, and graphemic cues, but these were gradually reduced as participants' naming abilities improved (following [50]). For each condition, participants received two one-hour therapy sessions per day for three consecutive days, with tDCS administered for the first 20 minutes of each session. At odds with Kang et al.'s and Rosso et al.'s findings, anodal rather than cathodal stimulation resulted in a significantly higher average percentage of correct, noncued naming of trained objects, with the effects being still evident two weeks after therapy. For the cathodal condition, although there was a significant improvement in naming compared to sham immediately after training, this positive effect was not maintained at the two-week follow-up. One key difference between this study and those of Kang et al. and Rosso et al., which could account for the discrepant results, is the location of stimulation. The expressive language functions associated with Broca's area are strongly left lateralized; however, the lexical-semantic functions associated with Wernicke's area are less, with the right Wernicke's homologue proposed to play a role in normal language processing (see e.g., [50]). As such, while a reduction of activation in Broca's homologue via cathodal stimulation may help restore left hemisphere functional dominance, leading to beneficial gains in naming performance, enhanced activation of the right Wernicke's homologue may help this region to better functionally compensate for the damaged left hemisphere, consistent with the findings of Menke et al. [50].

In summary, to date, a trio of studies have directly explored the effects of applying tDCS to the right hemisphere on noun naming ability with conflicting results. Both Kang et al.'s [98] and Rosso et al.'s [99] findings indicating that cathodal tDCS can improve naming ability are in line with previous TMS studies, while Flöel et al.'s [89] support for anodal rather than cathodal stimulation is consistent with a positive role for posterior right hemisphere activation in naming in some patients. Alongside varying patient characteristics, there are a number of differences between studies that may account for these discrepancies in results. For instance, Kang et al. and Rosso et al. chose more anterior stimulation sites, and the intervention protocols differed between all three studies. The current used was also stronger in Kang et al.'s study than in the two other studies. Further research is needed to clarify the effects of anodal and cathodal stimulation to anterior and posterior regions of the right hemisphere for participants with differing aphasic and lesion profiles and to directly compare the effects of right with left hemispheric stimulation.

### 4.3. Bilateral Stimulation

Lee et al. [100] investigated the added benefits of bilateral stimulation over unilateral stimulation. In their study, 11 aphasic individuals (six nonfluent and five fluent) received two 30-minute sessions of 2 mA tDCS. In one session, anodal tDCS over the left IFG was applied with concurrent sham stimulation over the right IFG. In the other session, simultaneous anodal tDCS over the left IFG and cathodal tDCS over the right IFG were applied, with the order of sessions counterbalanced across participants. During both sessions, reference electrodes were placed over the ipsilateral buccinator muscles. Speech and language therapy (involving picture naming and short paragraph reading) was provided during the last 15 minutes of stimulation of each session. Participants were tested immediately before and after each type of stimulation. Results showed that correct object picture naming scores on the short version of the Korean-BNT [109] increased significantly following both unilateral and bilateral stimulations. Only bilateral stimulation led to significant decreases in mean reaction time, although a nonsignificant reduction in mean reaction time was also noted following unilateral stimulation. In addition to changes in single object naming ability, Lee et al. measured pre- and postintervention verbal fluency in terms of the number of syllables produced during a picture description task. However, neither type of stimulation had any significant effects on this measure. Lee et al.'s findings suggest that bilateral left excitatory and right inhibitory stimulation of the IFG may be more effective than left excitatory IFG stimulation alone in improving confrontation object naming performance, yet they did not carry out any follow-up testing to check for longevity of the treatment effect. Nor did they include a sham condition. Furthermore, participants received only 15 minutes of speech and language therapy in each condition. This limited amount of input may, in part at least, explain why Lee et al. failed to support previous results reported by Fridriksson et al. [93] and Fiori et al. [94] who both found that unilateral anodal stimulation to the left hemisphere significantly reduced object naming reaction time following five 20-minute therapy plus tDCS sessions.

More recently, Manenti et al. [101] administered simultaneous bilateral stimulation to a 49-year-old woman with mild nonfluent aphasia for 25 minutes every weekday for four weeks. While stimulation was delivered offline in this study, each tDCS application was immediately followed by 25 minutes of semantic phonological action naming therapy (which required the participant to repeat the name of each verb three times and answer a series of questions regarding its semantic and phonological attributes), with the rationale that the neurostimulation may prime the resting language network for subsequent learning. The electrode montage used was similar to that adopted by Lee et al. [100], with anodal stimulation directed at the left dorsolateral prefrontal cortex and cathodal stimulation directed at the same area in the right hemisphere. The authors subsequently assessed the effects of the intervention program on a wide range of outcome measures. Results showed posttherapy gains in naming both treated and untreated verbs, indicating some degree of generalization, although the effects were greater for treated items. The percentage of correctly named verbs was unrelated to psycholinguistic characteristics such as frequency and number of syllables. Contrary to Lee et al.'s findings, Manenti et al.'s intervention program resulted in improvements in the participant's phonemic fluency, as well as her self-reported quality of life. Crucially, many of these effects were still evident at the 24- and 48-week follow-up periods, demonstrating the potential long-term benefits of tDCS-enhanced speech and language therapy programs.

There are a number of noteworthy features of Manenti et al.'s methodology that could be adopted in future research, such as their use of a diverse and extensive range of outcome measures, the length of their follow-up, and the provision of individualized therapy for their participant's verb naming deficit. However, the results generated in this study pertain to only a single individual with relatively mild language impairments, meaning that one cannot attempt to generalize the findings to the wider aphasic population. Moreover, the absence of a sham condition means that it is unclear what proportion of the observed gains can be attributed to tDCS relative to the contribution of the large number of therapy sessions provided. In addition, the participant received only one form of bilateral stimulation, making it impossible to state whether anodal stimulation to the left hemisphere or cathodal stimulation to the right hemisphere individually would actually have been more effective than both combined. It is also unclear whether concurrent (online) stimulation with therapy would also have had even greater positive effects.

The final study identified via the literature search describes three interrelated experiments involving a single individual with suspected crossed aphasia [102], a condition which occurs when a right handed individual presents with severe aphasia in the absence of structural damage to the left hemisphere [110]. Thus, the case studied by Costa and colleagues acquired her aphasia following a right middle cerebral artery (MCA) stroke, which resulted in damage to the right frontal, temporal, and parietal lobes. While it is also unclear from this case study whether combining bilateral stimulation with therapy would have enhanced the effects of stimulation (as again no concurrent therapy task was included), the authors investigated a wider range of bilateral electrode positions than either Lee et al. [100] or Manenti et al. (2013). Prior to their main experiments, Costa et al. carried out a brief pilot study, during which simultaneous anodal stimulation to Broca's area and cathodal stimulation to the right Broca's homologue were found to be more effective in increasing baseline scores on a noun and verb naming task than either simultaneous cathodal stimulation to Broca's area and anodal stimulation to the right Broca's homologue, or sham stimulation. Experiment 1 extended the findings of the pilot study by showing not only that simultaneous anodal tDCS to Broca's area and cathodal tDCS to the right Broca's homologue led to significantly higher naming scores but also that this effect was maintained for nine days. Experiments 2 and 3 followed the same procedure as Experiment 1, except that the electrodes were placed more posteriorly, in order to target Wernicke's area and the right Wernicke's homologue. In Experiment 2, anodal stimulation was delivered to the left hemisphere at the same time as cathodal stimulation to the right hemisphere, whereas Experiment 3 investigated the effects of the inverse electrode montage. Results showed that only the electrode arrangement in Experiment 3 led to significant increases in naming ability (this time maintained for six days after stimulation), indicating that, within this particular participant, the optimal simultaneous stimulation polarities for oral picture naming differed according to which cortical regions were targeted. Anodal stimulation to the intact (in this case, left) frontal lobe plus cathodal stimulation to the damaged (right) frontal lobe, and cathodal stimulation to the left temporal lobe plus anodal stimulation to the right temporal lobe were both linked to increased noun and verb picture naming ability. These findings are, however, difficult to interpret with respect to other studies, given that they pertain to just one individual with atypical language lateralization.

The three studies discussed above indicate that bilateral stimulation (comprising anodal tDCS to the left hemisphere and cathodal tDCS to the right hemisphere) may enhance naming ability in individuals with chronic anomia. Although Costa et al. [102] incorporated a range of bilateral stimulation montages in their case study, it is still unclear from the current studies whether bilateral stimulation is more effective than sham, unilateral left anodal, and/or unilateral right cathodal stimulation, and whether the effects hold true for larger groups of participants with typical left hemisphere language dominance.

## 5. Recommendations for Future Research

From the discussions above, it is clear that there is a growing body of evidence in support of the use of tDCS as an adjunct to enhance behavioral therapy in individuals with poststroke aphasia. However, it is also evident that this support is limited by its lack of systematicity and by the highly varied protocols used across studies [11, 13, 111]. As a consequence, a number of key issues regarding the methodological application of tDCS remain unresolved, including the individualization of electrode placement given different lesion locations, the exploration of a greater range of stimulation conditions and locations, and therapy delivery in relation to timing, tasks, targets, and outcome assessment.

Studies have varied regarding whether electrode placement was determined on a patient by patient basis, considering lesion size and location, or on a consistent target location basis, with the same key brain regions stimulated for all individuals. For example, Baker et al. [92] and Fridriksson et al. [93] used fMRI to determine electrode placement to ensure that stimulation targeted structurally intact cortex which had demonstrated the greatest activation associated with correct naming on a pretherapy naming task. However, in the majority of studies examined in the current review, a less individualized approach to electrode placement was used and, instead, electrodes were positioned over the same target brain regions in all participants, regardless of lesion location and extent, even when MRI scans showing precise lesion locations were available (e.g., [89, 95, 102]). A possible consequence of this more general approach is that certain participants may not have benefitted as anticipated from tDCS due to electrodes being placed over areas with insufficient viable underlying brain tissue. Some authors argue that precise placement is unnecessary as the effects of tDCS are generally fairly diffuse as a result of the size of active electrodes typically used (approximately 25–35 cm^2^) [74, 112]. Moreover, it is cheaper, simpler, and less demanding of patients if they are not required to undergo scanning prior to participation. Nevertheless, research has consistently highlighted the importance of recruitment of intact perilesional areas in poststroke recovery (e.g., [44]) and tDCS results have indicated that therapeutic benefits may be limited if stimulation does not target perilesional areas sufficiently close to patients' lesion sites [92]. Consequently, it would seem prudent to use scanning data, whenever available, to place electrodes where stimulation is believed to result in the best possible therapy outcomes.

Related to the issue of stimulation site, the current review found that, in the majority of the studies discussed, participants were given only one type of active stimulation to one region, while, in others, only one further condition (altering the polarity or location of stimulation) was included. This means that it is impossible to determine whether an alternative active stimulation condition would have led to even greater gains than those reported. The effects of cathodal tDCS to right contralesional areas remain generally underresearched compared to the effects of both anodal tDCS to the left hemisphere and TMS to the right pars triangularis. While one must caution against assuming that the effects of tDCS and TMS are equivalent [12], given the significant language benefits repeatedly observed after inhibiting right hemisphere activation using TMS, the role of cathodal tDCS to the right hemisphere warrants greater attention. Similarly, the effects of stimulation to posterior language regions (e.g., those surrounding Wernicke's area) are underrepresented relative to the effects on more frontal regions.

With the exception of Rosso and colleagues [99], who highlighted the differential effects of utilizing the same stimulation parameters with individuals with/out Broca's area intact, none of the reviewed studies explicitly compared the effects of stimulation on individuals with nonfluent and fluent aphasia following damage to different parts of the left hemisphere. Existing knowledge suggests that anodal stimulation applied to left frontal regions and/or cathodal stimulation applied to right frontal regions will yield the best results for individuals with nonfluent aphasia associated with frontal lesions and that anodal stimulation applied to left or right posterior regions will yield the best results for individuals with fluent aphasia associated with more posterior lesions. However, additional research is required to thoroughly investigate potential interactions between aphasia type and stimulation site/polarity. Furthermore, additional research should aim to clarify the relationship between aphasia severity and therapeutic effectiveness. In two studies [89, 91], the participants who showed the greatest gains from tDCS plus therapy were those with the most severe deficits. Fridriksson et al.'s [93] results support the notion that tDCS is more likely to increase naming speed than naming accuracy of patients with less severe aphasia, whose pretherapy accuracy may be near ceiling. It may be that tDCS has the potential to benefit individuals representing the full spectrum of symptom severities, but the optimum stimulation parameters for these individuals differ. This possibility should be addressed via more comprehensive research designs incorporating a range of participants and stimulation montages.

While several studies have suggested that tDCS can help to enhance naming for certain individuals in the absence of concurrent behavioral therapy [90, 91, 99], the majority of the studies indicate that combining tDCS with a therapy task leads to more consistent gains. The therapy tasks utilized vary across studies, making direct comparison impossible, although all tasks required participants to take an active role by matching stimuli, producing item names, or answering questions regarding items' properties. It may be the case that the particular therapy task is less important to the success of tDCS plus therapy interventions than the location and polarity of stimulation; however, this is another factor that could be explored in the future. The therapeutic protocols adopted by previous studies also differ in terms of the number of sessions, the length of any follow-up, and the outcome measures adopted. Regarding the frequency of tDCS plus therapy sessions, the majority of studies have incorporated fairly intensive and often extensive therapy schedules, with clients receiving stimulation every day for three to 20 days. As mentioned previously, this type of schedule can be difficult to maintain in clinical practice for various reasons [12]. Within the domain of behavioral language therapy, studies have found that both intensive and nonintensive anomia therapies may lead to similarly significant improvements in naming ability. Indeed, there is evidence that long-term retention may actually be greater when equal hours of therapy are distributed over five rather than two weeks [113]. Consequently, future research could investigate whether the observed beneficial effects of tDCS and speech and language therapy can be achieved using less frequent sessions, reducing the demands on clinicians and patients alike. On a related note, the longer that therapy effects remain evident, the less often any potentially time-consuming and costly repeat or “top up” treatment needs to be administered. Despite research with healthy adults indicating that beneficial effects of tDCS on cognitive abilities can remain significant for at least twelve months [64], many of the studies discussed above failed to investigate any possible lasting effects of intervention. When participants were tested following a posttreatment interval, other than Manenti et al.'s [101] notable case study and Vestito et al.'s small pilot study, the longest follow-up was four weeks after therapy. Further, larger studies involving much longer follow-ups are clearly required to investigate how long any significant outcomes following tDCS plus anomia therapy persist in the majority of individuals.

Predictably, given the scope of the literature search, the primary outcome measure in all of the above studies was unassisted confrontation naming of noun and/or verb pictures. In the majority of studies, only noun naming was examined, although Fiori et al. [95] revealed an interesting potential interaction between stimulation site and word class: anodal tDCS to Broca's area resulted in significantly better verb naming and anodal tDCS to Wernicke's area resulted in significantly better noun naming. The observation that anodal tDCS to frontal regions may particularly enhance verb naming is supported by Marangolo et al. [96] but not Vestito et al. [97]. Given the small number of studies and patients involved, more research involving within-participant designs is clearly indicated. Regardless of whether nouns, verbs, or both were considered, almost all studies looked only at improvements in naming treated items rather than the effects of therapy on naming both treated and untreated items. It is, of course, impossible to treat all words that individuals with anomia have difficulty with in therapy; therefore, it is crucial that therapies have the potential to generalize from treated to untreated items. Such generalization has been documented in the behavioral anomia therapy literature (e.g., [114]) and the small number of the existing tDCS studies to address generalization has suggested that stimulation plus therapy may lead to some increases in naming of untreated items [92, 101]. However, future research designs could further investigate the potential for significant generalization by incorporating testing of both treated and untreated items at baseline and all follow-up time points.

Additionally, within the field of aphasia rehabilitation, there is a general consensus that single noun and verb naming ability can be influenced by the psycholinguistic properties of the words involved, such as age of acquisition, frequency, familiarity, imageability, concreteness, length, typicality, and animacy (e.g., [115, 116]). As mentioned in Section 2, there is also a growing body of evidence to suggest that different cortical regions may be involved in naming words with certain properties [25, 27]. Given the apparent importance of psycholinguistic properties for naming, it is perhaps surprising to note that there is a current paucity of evidence regarding potential interactions between such variables and the observed effects of tDCS on confrontation naming ability. Several studies, which included treated and untreated word sets or a number of treated sets, explicitly stated that sets were matched on the basis of particular psycholinguistic variables. For example, Baker et al.'s [92] treated and untreated noun sets were matched for frequency (low/medium/high), semantic category, and word length. However, only one study [101] provided further discussion regarding which words benefited most from tDCS. In this study, Manenti and colleagues [101] found that psycholinguistic properties had no effects on verb naming in their study, although their findings pertain to a single case with mild aphasia. More detailed examination of the impact of psycholinguistic variables on the effectiveness of tDCS-based therapeutic interventions in the wider patient population is undoubtedly warranted.

Finally, it is important that statistically significant increases in picture naming performance translate into meaningful changes to patients' everyday communication [7, 117, 118]. Thus, while two existing studies assessed verbal fluency [100, 101], no studies to date have measured the potential effects of therapy on functional, real-life conversational abilities. Moreover, given the known adverse impact of aphasia on individuals' well-being and social interactions [2], it is perhaps surprising that the majority of previous studies (again with the exception of [101]) have also failed to include any outcome measures related to these factors. It is clear that ongoing research would benefit from the inclusion of a variety of outcome measures designed to assess the effects of tDCS plus anomia therapy intervention programs on functional communication and socioemotional factors.

### 5.1. Summary

While there is growing evidence that tDCS can enhance the effects of behavioral speech and language therapy for anomia, further research is required to segregate the effects of varying the polarity, site, timing, and frequency of stimulation in order to determine optimal tDCS parameters for maximal benefits. In particular, future studies should

1. consider the effects of tDCS on naming ability with concurrent speech and language therapy tasks as this approach seems to provide the most consistent gains;
2. utilize within-participants study designs, with individuals receiving sham stimulation as a control condition;
3. consider the effects of stimulation in the context of the patient's lesion site, stage of recovery, and behavioral profile/severity of anomia;
4. optimize electrode placement by exploiting neuroimaging data, using new head models that take into account the extent to which individual lesions affect current flow;
5. consider systematically the polarity (anodal versus cathodal) and laterality (left and/or right hemisphere) of stimulation to determine which electrode montage leads to the greatest improvements in picture naming ability;
6. examine directly the effects of tDCS in relation to both word class (nouns versus verbs) and the psycholinguistic properties of targeted items;
7. vary the number and frequency of tDCS plus therapy sessions to determine whether similar gains can be achieved via less intensive treatment protocols;
8. explore the longevity of tDCS effects by incorporating postintervention follow-ups greater than four weeks;
9. highlight any potential generalization by assessing the effects of tDCS on naming both treated and untreated items;
10. incorporate a more extensive range of outcome measures to assess not only accuracy and speed of confrontation naming, but also effects on connected speech tasks and quality of life measures. This would facilitate fuller understanding of the range of potential gains from tDCS plus therapy intervention programs.

## 6. Conclusion

Successful picture naming is a complex task that relies on multiple, interconnected brain regions, many of which are left lateralized in healthy individuals. Anomia arises when parts of the normal naming network are damaged, for example, by a stroke. Long-term recovery from poststroke anomia is facilitated by a number of cortical mechanisms and, in particular, by spontaneous and/or therapy-induced relateralization of language skills to the left hemisphere. Behavioral speech and language therapy can promote relateralization; however, research increasingly supports the use of neurostimulation techniques in lieu of, or in conjunction with, naming therapy to aid this process. Applying inhibitory TMS to the right Broca's homologue can significantly enhance naming performance in individuals with chronic aphasia, both as a standalone approach or when immediately followed by behavioral therapy. There is also limited evidence that administering excitatory TMS to left hemisphere language areas followed by such therapy produces similar benefits. However, tDCS offers increased patient comfort and safety over TMS and, consequently, may be the more useful therapeutic tool. Studies have revealed significant effects of tDCS and concurrent speech and language therapy on the naming ability of stroke survivors, in particular demonstrating that anodal (excitatory) stimulation to the left hemisphere and/or cathodal (inhibitory) stimulation to the right hemisphere can significantly increase naming accuracy and speed. To determine optimal therapeutic protocols, future research should incorporate more comprehensive designs in terms of polarity, site, frequency, and timing of stimulation for patients with different lesion sites at different stages of language recovery. A greater number of well-designed studies could one day help to translate the potential of tDCS as an adjunct to behavioral speech and language therapy into clinical practice, resulting not only in increased naming ability but also in improved quality of life for those with chronic anomia.

## Figures and Tables

**Table 1:** tDCS studies of naming ability of individuals with chronic poststroke aphasia. Images are supplied to illustrate key aspects of the protocol. Ovals represent stimulation site, with size reflecting electrode size. Red ovals represent anodal stimulation, blue ovals represent cathodal stimulation, and grey ovals represent sham stimulation. Symbols on the ovals indicate target site; symbols alone indicate reference electrodes.

Study	tDCS protocol	Number of participants	Months after stroke	Aphasia profile	Concurrent therapy	Outcome measures	Initial results (mean values)	Length of follow-up
Left hemisphere								

Monti et al. 2008 [90]	2 mA, 10 mins, single sessions, electrodes 35 cm^2^ *Experiment 1* At least a week between anodal or/and cathodal and sham	8 in total			None	Noun picture naming accuracy and reaction time	Naming accuracy increased significantly (+33.6%) following cathodal stimulation but not after anodal or sham stimulation There were no significant changes in reaction time following anodal, cathodal or sham stimulation	N/A
	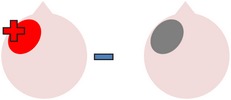	4 + 2 also cathodal						
	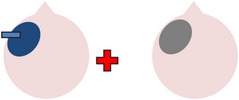	4 + 2 also anodal	24–96	4 × Broca's 4 × Global				
	2 months later *Experiment 2* Time between cathodal and sham not reported 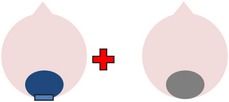 Reference electrode on contralateral shoulder.				None	Noun picture naming accuracy and reaction time	There were no significant changes in either naming accuracy or reaction time following cathodal or sham stimulation	N/A

Volpato et al. 2013 [91]	2 mA, 20 minutes × 5 days for 2 weeks, electrodes 35 cm^2^ Time between anodal and sham not reported 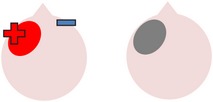	8	6–126	2 × Anomic 1 × Broca's 1 × Conduction 1 × Transcortical motor 1 × Transcortical sensory 2 × Wernicke's mild- moderate	None	Noun and verb picture naming accuracy and reaction time	Anodal tDCS significantly improved verb picture naming accuracy (+184.62%) and reduced reaction time (−32.68%) for only 1 ppt, with the most severe anomia There were no significant effects of stimulation on noun picture naming accuracy and speed	N/A

Baker et al. 2010 [92]	1 mA, 20 mins × 5 days for 1 week, electrodes 25 cm^2^ At least one week between anodal and sham 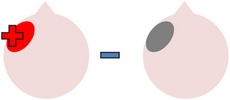	10	10–242	6 × Anomic 4 × Broca's Wide ranging severity of aphasia	Computerized noun naming therapy	Noun picture naming accuracy Treated and untreated items	Anodal tDCS significantly improved the naming accuracy of treated items and numerically increased (from 27.3 to 40/50 after treatment) the number of untreated items named correctly	1 week: the significant effect of anodal stimulation was maintained and the number of untreated items named correctly increased further (42/50, still n.s.)

Fridriksson et al. 2011 [93]	1 mA, 20 mins × 5 days for 1 week, electrodes 25 cm^2^ 3 weeks between anodal and sham 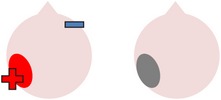	8	10–150	Fluent	Computerized noun naming therapy	Noun picture naming reaction time Treated and untreated items	Anodal tDCS significantly reduced reaction times (−455.57 ms) for 7/8 ppts on treated items versus sham tDCS (−281.17 ms) There were no significant effects of stimulation on untreated items	3 weeks: all 8 ppts now showed reduced reaction times for treated items after anodal tDCS (−430.6 ms) and not after sham tDCS (−265.86 ms)

Fiori et al. 2011 [94]	1 mA, 20 mins × 5 days for 1 week, electrodes 35 cm^2^ One week between anodal and sham 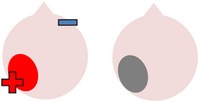	3	21–71	Nonfluent (1 × mild, 1 × moderate, 1 × severe)	Computerized noun naming therapy	Noun picture naming accuracy and reaction time Treated items only	Naming accuracy significantly increased (+21% more than sham) and reaction time significantly reduced following anodal tDCS rather than sham tDCS (1486 ms versus 1763 ms)	1 and 3 weeks (only 2/3 ppts): some reduction in naming accuracy from the end of therapy to 1 week follow-up (still significant) effects on reaction times maintained

Fiori et al. 2013 [95]	1 mA, 20 mins × 5 days for 1 week, electrodes 35 cm^2^ Six days between anodal Wernicke's, anodal Broca's and sham, one month between noun cycle and verb cycle 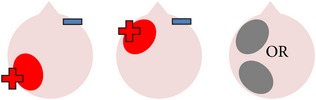	7	7–84	Nonfluent with noun and verb retrieval deficits	Computerized noun and verb naming therapy	Noun and verb picture naming accuracy Treated items only	Anodal tDCS to Broca's area significantly improved verb naming accuracy (Broca's versus Wernicke's = +24%, Broca's versus sham = +22%). Anodal tDCS to Wernicke's area significantly improved noun naming accuracy (Wernicke's versus Broca's = +17%, Wernicke's versus sham = +24%)	1 and 4 weeks: significant effects of Broca's stimulation on verb naming and of Wernicke's stimulation on noun naming persisted

Marangolo et al. 2013 [96]	1 mA, 20 mins × 5 days for 1 week, electrodes 35 cm^2^ Six days between anodal Wernicke's, anodal Broca's and sham 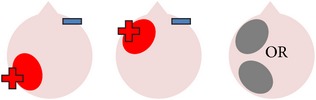	7	7–84	Nonfluent with verb retrieval deficits	Computerized verb naming therapy	Verb picture naming accuracy Treated items only	Anodal tDCS to Broca's area significantly improved verb naming accuracy. (% correct responses: Broca's = 33% Wernicke's = 24% Sham = 23%)	1 and 4 weeks (only 6/7 ppts): effects maintained

Vestito et al. 2014 [97]	1.5 mA, 20 mins × 5 days for 2 weeks, electrodes 25 cm^2^ Anodal one hour after sham 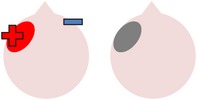	3	20–64	2 × nonfluent (1 × high, 1 × very high severity) 1 × Anomic (moderate severity)	Noun and verb naming therapy Therapy task difficulty was increased for the second week (different item set with increased number of lower frequency words)	Noun and verb picture naming accuracy Treated items only Boston Naming Test (BNT), Aachen Aphasia Test (AAT) (naming, oral/written comprehension)	Anodal stimulation significantly increased the number of items correctly named from baseline, with initial increases following the first session and further increases over the remaining sessions each week for ppt 1 (week 1 15/24/28, week 2 8/24/30) and ppt 3 (26/30/35, week 2 27/31/36), and for week 2 for ppt 2 (16/22/26) Therapy task difficulty was unrelated to naming outcomes Anodal stimulation increased % correct responses for all ppts on the BNT (ppt 2 and ppt 3 n.s.) and AAT (ppt 3 n.s.)	4, 8, 12, 16, and 21 weeks: effects on number of correct responses persisted significantly for all ppts to 16 weeks and persisted up to 21 weeks (n.s.) % correct responses on the AAT and BNT persisted significantly up to 12 weeks and persisted up to 21 weeks (n.s.)

Right hemisphere								

Kang et al. 2011 [98]	2 mA, 20 mins × 5 days for 1 week (starting 10 minutes into each 30-minute training session) electrodes 25 cm^2^ One week between cathodal and sham 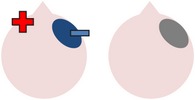	10	6–180	2 × Anomic 3 × Global 4 × nonfluent 1 × Transcortical motor	Individually tailored computerized noun retrieval therapy	Noun picture naming accuracy (including % cued responses) and reaction time on Korean version of BNT	Trend for increased naming accuracy following cathodal tDCS versus sham (*p* = 0.058)	1 hour: trend still apparent

Rosso et al. 2014 [99]	1 mA, 15 mins × single sessions, electrodes 35 cm^2^ Two hours between cathodal and sham 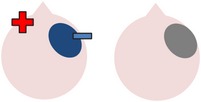	25	>3 (mean = 15)	Picture naming deficits Range of severity of aphasia 11 ppts with lesions involving Broca's area (B+), 14 with lesions not involving Broca's area (B−)	None	Noun picture naming accuracy (calculated as a function of the number of correct and partially correct (e.g., containing one phonemic error) responses)	Naming accuracy of B+ ppts increased significantly following cathodal tDCS, naming accuracy of 13/14 of B− ppts decreased or remained the same following cathodal stimulation Greater improvements in naming were also associated with greater integrity of the arcuate fasciculus	N/A

Flöel et al. 2011 [89]	1 mA, 20 mins × twice per day for 3 days (at start of each training hour), electrodes 35 cm^2^ 3 weeks between anodal, cathodal and sham 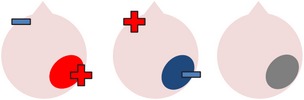	12	14–260	2 × Anomic 7 × Broca's 1 × Global 1 × Wernicke's 1 × not classified	Computerized noun naming therapy involving a decreasing cueing hierarchy	Noun picture naming accuracy Treated items only	All conditions resulted in increased naming ability (= 83%), but anodal tDCS led to significantly greater improvements than cathodal or sham stimulation Ppts with more severe anomia showed the greatest therapy gains	2 weeks: effects persisted

Bilateral								

Lee et al. 2013 [100]	2 mA, 30 mins, single sessions, electrodes 25 cm^2^, therapy given during last 15 minutes of stimulation >24 hours between anodal + sham and bilateral conditions Reference electrodes were placed over the ipsilateral buccinator muscles 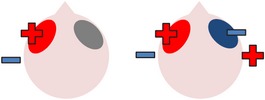	11	6+	4 × Broca's 2 × Transcortical motor 5 × Anomic	Picture naming and reading short paragraphs	Noun picture naming accuracy and reaction time on Korean version of the BNT Verbal fluency	Naming accuracy significantly increased in both conditions Reaction time decreased in both conditions, but this was only significant for the bilateral stimulation condition Stimulation had no effect on verbal fluency	N/A

Manenti et al. 2015 [101]	2 mA, 25 minutes × 5 days for 4 weeks, electrodes 35 cm^2^ Anodal and cathodal delivered simultaneously 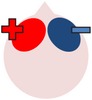	1	8	Mild nonfluent	None 25 minutes of semantic-phonological therapy given directly after each stimulation session	Nonverbal reasoning, verbal fluency, Aachen Aphasia Test (AAT), Battery for the Analysis of Aphasia Deficits (BADA), Stroke and Aphasia Quality of Life Scale-39 (SAQOL-39), noun and verb picture naming accuracy Treated and untreated items	There were a number of significant changes at 4 weeks after stimulation Phonemic fluency: significant increase SAQOL-39: significant increases in psychosocial/mood and communication scales Verb naming: significant increases in % named correctly (treated and untreated items) and significant decreases in number of “circumlocution” and “replacement with noun” errors	12, 24, and 48 weeks of phonemic fluency: further increases at 48 weeks SAQOL-39: effects on psychosocial/mood scale maintained at 24 weeks and on communication scale at 48 weeks Verb naming: effects on % named correctly maintained at 48 weeks and effects on error type maintained at 24 weeks

Costa et al. 2015 [102]	1 mA, 20 minutes, electrodes 16 cm^2^ *Pilot study* 3 single sessions, one week between conditions 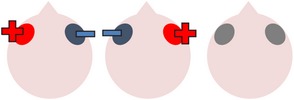				None	Scores on a noun and verb naming task (calculated as a function of correct responses without cues and with one/two letter phonological cues)	Naming scores were significantly higher than baseline following anodal left/cathodal right stimulation than following either cathodal left/anodal right or sham stimulation (*p* = 0.017) There was no significant difference between noun and verb naming	N/A
	1 month later *Experiment 1* *20 minutes × 5 days for 2 weeks* 9 days between simultaneous and sham 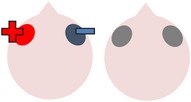	1	30	Severe nonfluent Possible crossed aphasia	None	Scores on the noun and verb naming task	Naming scores were significantly higher than baseline following active than following sham stimulation (*p* < 0.05) There was no significant difference between noun and verb naming	Scores taken every three days after stimulation; effect maintained for 9 days
	4 months later *Experiment 2* *20 minutes × 5 days for 2 weeks* 9 days between simultaneous and sham 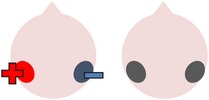				None	Scores on the noun and verb naming tas	There was no significant difference in naming scores following active or sham stimulation There was no significant difference between noun and verb naming	N/A
	4 months later *Experiment 3* *20 minutes × 5 days for 2 weeks* 9 days between simultaneous and sham 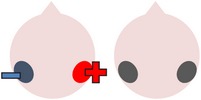				None	Scores on the noun and verb naming task	Naming scores were significantly higher than baseline following active than following sham stimulation (*p* < 0.05) There was no significant difference between noun and verb naming	Scores taken every three days after stimulation; effect maintained for 6 days
